# Massive Radicular Cyst in the Maxillary Sinus as a Result of Deciduous Molar Tooth Pulp Necrosis

**DOI:** 10.1155/2020/8837706

**Published:** 2020-08-04

**Authors:** Damian Chybicki, Małgorzata Lipczyńska-Lewandowska, Monika Ratajek-Gruda, Anna Janas-Naze

**Affiliations:** Department of Oral Surgery, Central Clinical Hospital, Medical University of Lodz, Poland

## Abstract

The article describes a rare case of radicular cyst associated with deciduous right upper molars in a 5 y.o. male patient. The cyst developed asymptomatically in the maxillary sinus, thus causing considerable displacement of both premolar germs. Due to the severity of surgery, the patient was treated under general anesthesia in a one-day surgery system with use of the enucleation method. The extent of the lesion results in necessity of removal of the second premolar germ. Early diagnosis of the lesion would have resulted in a more conservative treatment plan. The purpose of this article is to lay emphasis on the pedodontist's role in early diagnosis of such lesions.

## 1. Introduction

Radicular cysts are the most common cysts of the jaws, with occurrence of 7 to 54% of all the cysts in permanent dentition, whereas radicular cysts of deciduous dentition are extremely rare (0.5 to 3.3%) due to the natural course of milk tooth exfoliation [1, 2]. The apexes of these teeth are commonly involved in the cyst. Most radicular cysts develop asymptomatically and are accidentally discovered during a radiological examination performed for another reason [1]. Radicular cysts arise from the epithelial remnants of the periodontal ligament as a result of inflammation, migration, and infiltration of inflammatory cells such as leukocytes and monocytes, which is an effect of pulp necrosis [2]. This suggests that caries is the most important etiological factor of this disease entity. In cases of symptomatic inflammation of the pulp of milk teeth, pulp therapy is introduced to save the tooth for as long as it is possible, preferably until its natural replacement by the permanent tooth. Complications of this treatment may include cyst formation, delayed deciduous tooth resorption, or permanent tooth enamel defects. Developing cysts can lead to displacement of permanent tooth germs, disorders of eruption, or even partial destruction. [2]. Unfortunately, inflammation of the pulp leading to its necrosis can also be asymptomatic, and therefore, the developing pathology (a cyst) may remain unnoticed and reach considerable sizes.

Among the methods of treating this type of cysts, it is preferable to enucleate the lesion with an attempt to preserve germs of permanent teeth, which is not always possible [2]. With this method, the entire lesion is removed and it can be histopathologically examined. Correct eruption of permanent teeth is usually observed, even when their germs have been dislocated [2].

In radiographic images, radicular cyst appears as a round or oval unilocular radiolucency in the area of the apexes of the causal teeth, with a radiopaque sclerotic margin, that may be blurred in cysts with high growth dynamics. Standard radiographic images show only two dimensions, so to assess the extent of the bone and soft tissue defect accurately, in three dimensions (sagittal, coronal, and axial), it is advisable to perform cone beam computed tomography [1]. Cone beam computed tomography, which was introduced specifically for dentomaxillofacial imaging, is currently widely used in dentistry, e.g., in endodontics, oral surgery, or dental implantology, taking into account its advantages such as reducing the effective radiation dose, lower costs, and the possibility of image processing [3].

## 2. Case Report

**Figure 1:**
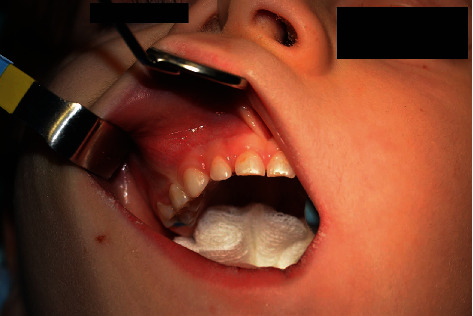
Intraoral view on the operative region.

A 5-year-old healthy patient was referred by his general dental practitioner to the Department of Oral Surgery, Medical University of Lodz, for a consultation of the edema in the buccal vault of the mouth in the area of teeth 54 and 55. The lesion was asymptomatic, and parents of the patient did not notice when it began. The extra oral examination showed no pathologies, whereas the intraoral examination resulted in disclosure of protuberance in the area of apexes of right upper deciduous molars with no symptoms of inflammation—mucosa was pink, shiny, and smooth (Figure 1).

**Figure 2:**
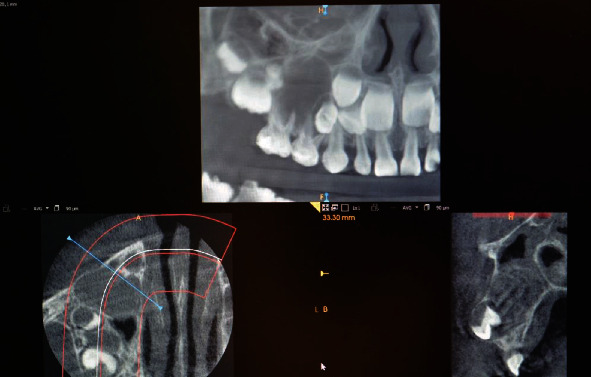
CBCT scans (GXDP-800, OP300-1, Gendex Dental Systems, 2017, Medical University of Lodz, Poland. Software: InVivoDentalViewer 5.1, Anatomage, Milan, Italy).

Teeth 54 and 55 had restorations on their occlusal and distal surfaces and gave no response to pulp sensibility testing. The computed tomography was performed, and it revealed a vast lesion above the teeth in described location and maxillary sinus with displacement of premolar germs (Figure 2).

Based on clinical examination and computed tomography evaluation, the initial diagnosis of radicular cyst was made and a treatment plan was established. The diagnosis and treatment plan including removal of teeth 54 and 55, enucleation of cyst, and intraoperative evaluation of possibility of saving premolar germs were presented to the patient's parents, and all surgical permissions were collected. The preoperative recommendations, medications, and laboratory blood tests were ordered (complete blood count, APTT) and the surgery scheduled.

**Figure 3:**
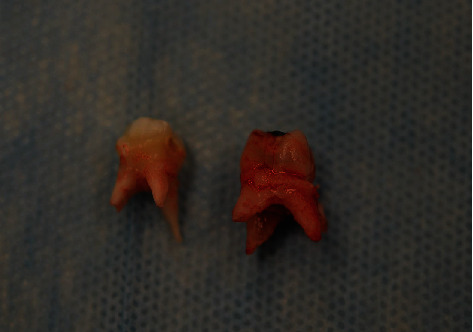
Teeth 54 and 55 removed.

**Figure 4:**
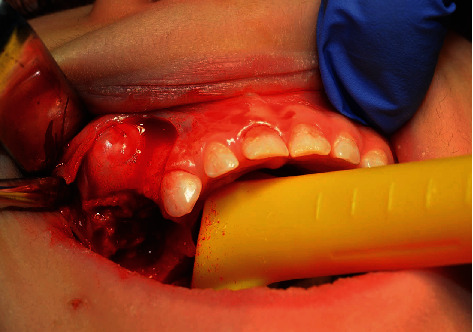
Enucleation.

**Figure 5:**
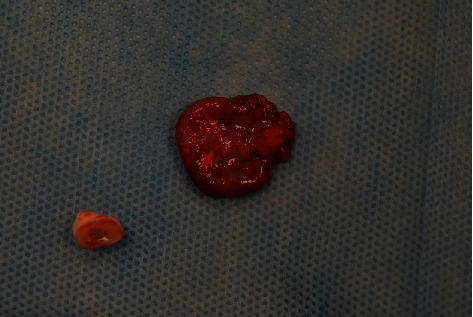
Cyst and tooth 15 germ.

Under general anesthesia (short-term intravenous anesthesia with use of combination of 50 mg of propofol and 20 *μ*g of fentanyl with monitoring of saturation and heart rate), teeth 54 and 55 were removed (Figure 3); the flap was designed with vertical releasing incision in the area of tooth 53 with scalpel blade no. 15 (Swann-Morton, Sheffield, England, 2018) and reflected with Molt 9 periosteal elevator (Kohler Medizintechnik, Stockach, Germany, 2019). The cyst was enucleated (Figure 4) with the germ of tooth 15 which was involved into the lesion, pushed beyond the bone, to the maxillary sinus (Figure 5), so the prognosis was poor.

The flap was repositioned, and simple interrupted sutures were taken (Novosyn 4/0, B. Braun, Germany, 2019). The enucleated material was conserved in 10% formalin solution and sent for histological examination. The patient regained consciousness in the recovery room and was discharged home. Postoperative antibiotic (150 mg of clindamycin every 8 hours) and analgetic (150 mg of ibuprofen depending on the needs, but not more than three times a day) were prescribed.

Follow-up examination was performed the day after operation. Proper healing of the wound was observed, along with mild edema of the right cheek and slight pain. Sutures were left for spontaneous dissolution.

Three weeks after surgery, the wound was completely healed. The patient did not report any complaints. The result of histological examination as radicular cyst was presented to the patient's parents. These results illustrated a cystic lumen, lined with nonkeratinized stratified squamous epithelium and partially with pseudostratified ciliated columnar epithelium. In the connective tissue wall, among inflammatory cells, cholesterol crystals were observed.

## 3. Discussion

A radicular cyst is usually associated with carious, nonvital, or fractured tooth [4, 5]. In addition, some authors pay attention also to root canal-treated deciduous teeth using gutta-percha as a root canal filling or formocresol as a medication under temporary filling [6, 7]. Despite the fact that it is mostly connected with permanent teeth, it sometimes develops also in the area of deciduous teeth.

In general, radicular cysts develop progressively and asymptomatically, finally revealing itself with subsequent displacement of adjacent teeth and malocclusion or bony expansion [6]. This is usually the reason why dentists decide to perform radiological examination. It is extremely important to diagnose such pathologic lesions as early as possible to ensure adequate treatment to protect the normal growth of the jawbone and teeth. Taking into account the fact that inflammation of the pulp and periapical tissues in the primary dentition has a greater tendency to drainage than in the permanent dentition (this is related to bone mineralization), symptoms may occur later, which makes it even more difficult to recognize the problem early enough [1, 2].

Most radicular cysts in primary dentition are associated with mandibular molars with extensive dental caries [2]. That does not mean that we can neglect proper examination in the area of maxillary molars, which is shown by this case report, because also in this unusual location such lesions may occur. Periapical radiolucencies of primary teeth may be misdiagnosed as a dentigerous cyst of the permanent successor or periapical granuloma [2]. For this reason, preoperative misdiagnosis is an additional problem. Accurate analysis of the position of the permanent tooth germ during radiological examination, followed by intraoperative examination and at the final stage also histopathologic examination, is crucial for the proper management of the lesion and avoiding unnecessary extractions of permanent tooth germs [8].

As mentioned earlier, complete enucleation of the cyst and preservation of the permanent successor teeth are recommended as the most suitable treatment option in these cases [2]. The present report showed that preservation of the permanent teeth is not always possible which depends on the extent of the lesion and tooth germ location. Alternative treatment that can be used to manage massive cysts of the jaws includes marsupialization of the cystic lesion and using an appliance with projection for decompressing the lesion [2, 7]. The postoperative period is very demanding in this case which excludes the use of this method in small, often-noncooperating children, as was in the described case in [7]. The undeniable advantage of this method is its more conservative nature, increasing the chance of saving permanent tooth germs, but the absolute condition of its implementation is full patient cooperation.

Given the low incidence of radicular cysts in primary dentition, this case report informs about the possibility of extensive lesions of this type forming in the area of primary teeth not only endodontically treated but also with restorations remaining in close proximity to the pulp of these teeth, which should alert dentists during follow-up visits, even when teeth remain asymptomatic.

The premature primary tooth loss is a serious problem that, if neglected, can affect the life of maturing children. In the presented case, the 5 y.o. patient had both deciduous right maxillary molars removed due to radicular cyst that was developing in this area. The premature loss of deciduous molars before the eruption of the first permanent molars causes significant mesial movement of the first permanent molars, which are mesialized at the moment of the eruption, using the available space and thereby reducing the arc length. To avoid loss of space when teeth are lost, there is possibility to place a space maintainer [9]. In this case, occlusal disorders that result from premature loss of deciduous molars and second premolar germ were so significant that this simple way of treatment was useless, and highly specialized orthodontic treatment was recommended to guide the swelling of permanent teeth.

The present report showed a radicular cyst. Other cysts that can be found in oral cavity in pediatric age are lateral periodontal cysts [10], mucoceles [11], glandular odontogenic cysts [12], and solitary bone cysts [13]. None of these lesions is common. Lateral periodontal cysts are odontogenic cysts that develop on lateral surfaces of tooth roots. Teeth associated with these lesions are vital, which is the main feature that distinguishes them from radicular cysts. The cyst can be also managed through enucleation with GBR to enhance the healing process [10]. Mucocele is a salivary gland mucous cyst most frequently located on the lower lip. This lesion is also usually asymptomatic; it can interfere with respiration and feeding, especially in infants. The complete incision of the lesion with blade or diode laser is a sufficient treatment [11]. Glandular odontogenic cysts are associated with unerupted teeth. Despite the fact that these lesions are usually asymptomatic, they can be destructive for surrounding tissues. The diagnosis can be challenging due to their nonspecific microscopic features. Among the methods of treatment are not only conservative surgery but also marginal or partial resection [12]. The solitary bone cyst is considered pseudocyst due to the lack of epithelial lining. The etiopathogenesis still remains controversial, but trauma is considered an important factor in their development. The decompression technique with needles can be used as a treatment for reducing the size of the lesion, without enucleation, which is unique comparing to previous lesions [13]. The oral cavity is a specific place in the human body that requires great care and caution from the dentist due to the variety of pathological lesions and difficulties in their differentiation.

The only alternative option for treatment under local anesthesia is general anesthesia which seems to be a necessary tool that enables proper treatment regardless of the patient's age. Sometimes deep sedation or general anesthesia is a treatment of choice (and the only possible option) because of the patient's extensive treatment needs, acute situational anxiety, uncooperative age-appropriate behavior, limited cognitive functioning, physical disability, or medical conditions that require deep sedation or general anesthesia to complete dental treatment in a safe and humane fashion [14]. The aim of using general anesthesia is to restore optimal oral health in a single visit with all diligence, without worries about young patient cooperation [15].

## 4. Conclusions

The role of general dental practitioners and pediatric dentists in detecting pathological lesions that may develop asymptomatically for a long time should be emphasized, as early detection of the lesion allows the use of less radical methods of treatment, which is crucial in young patients. For this purpose, periodic radiographic images of treated deciduous teeth with a diagnosis of deep caries or pulp inflammation should be taken. Early diagnosis is important to avert adverse effect to the underlying permanent teeth.
